# Optimal Sequencing of Deceased Donor and Live Donor Kidney Transplant Among Pediatric Patients With Kidney Failure

**DOI:** 10.1001/jamanetworkopen.2021.42331

**Published:** 2022-01-06

**Authors:** Bryce A. Kiberd, Amanda Vinson, Philip D. Acott, Karthik K. Tennankore

**Affiliations:** 1 Department of Medicine, Dalhousie University, Halifax, Nova Scotia, Canada; 2 Department of Pediatrics, Dalhousie University, Halifax, Nova Scotia, Canada

## Abstract

**Importance:**

In the US, live donor (LD) kidney transplant rates have decreased in pediatric recipients. Pediatric patients with kidney failure will likely need more than 1 kidney transplant during their lifetime, but the optimal sequence of transplant (ie, deceased donor [DD] followed by LD or vice versa) is not known.

**Objective:**

To determine whether pediatric recipients should first receive a DD allograft followed by an LD allograft (DD-LD sequence) or an LD allograft followed by a DD allograft (LD-DD sequence).

**Design, Setting, and Participants:**

This decision analytical model examined US pediatric patients with kidney failure included in the US Renal Data System 2019 Report who were waiting for a kidney transplant, received a transplant, or experienced graft failure.

**Interventions:**

Kidney transplant sequences of LD-DD vs DD-LD.

**Main Outcomes and Measures:**

Difference in projected life-years between the 2 sequence options.

**Results:**

Among patients included in the analysis, the LD-DD sequence provided more net life-years in those 5 years of age (1.82 [95% CI, 0.87-2.77]) and 20 years of age (2.23 [95% CI, 1.31-3.15]) compared with the DD-LD sequence. The net outcomes in patients 10 years of age (0.36 [95% CI, −0.51 to 1.23] additional life-years) and 15 years of age (0.64 [95% CI, −0.15 to 1.39] additional life-years) were not significantly different. However, for those aged 10 years, an LD-DD sequence was favored if eligibility for a second transplant was low (2.09 [95% CI, 1.20-2.98] additional life-years) or if the LD was no longer available (2.32 [95% CI, 1.52-3.12] additional life-years). For those aged 15 years, the LD-DD sequence was favored if the eligibility for a second transplant was low (1.84 [95% CI, 0.96-2.72] additional life-years) or if the LD was no longer available (2.49 [95% CI, 1.77-3.27] additional life-years). Access to multiple DD transplants did not compensate for missing the LD opportunity.

**Conclusions and Relevance:**

These findings suggest that the decreased use of LD kidney transplants in pediatric recipients during the past 2 decades should be scrutinized. Given the uncertainty of future recipient eligibility for retransplant and future availability of an LD transplant, the LD-DD sequence is likely the better option. This strategy of an LD transplant first would not only benefit pediatric recipients but allow DD kidneys to be used by others who do not have an LD option.

## Key Points

**Question:**

If a pediatric recipient has a live donor (LD) kidney option available, should they opt for this transplant first or opt for a deceased donor (DD) transplant first and save the LD option for later if required?

**Findings:**

In this decision analytical model, opting for an LD transplant first most often provides more remaining life-years. However, opting for a DD transplant first and saving an LD for retransplant after the DD transplant fails may result in a similar number of remaining life-years for recipients aged 10 to 14 years while the LD remains available.

**Meaning:**

With the contemporary trend of proportionately fewer LD kidney transplants for pediatric recipients, programs should promote LD kidney transplant as the first option for pediatric recipients.

## Teaser

This decision analytical model assesses conditions that favor a deceased donor transplant followed by a live donor transplant sequence or vice versa in pediatric patients with kidney failure.

## Introduction

During the past 2 decades, live donor (LD) kidney transplant rates in the US have decreased, but of greater concern, the decrease has been uneven.^1^ Comparing 2001 through 2005 with 2015 through 2019, there were 41% fewer pediatric (aged <18 years) LD kidney recipients, with a decrease from 2115 to 1242.^1^ In comparison, the number of deceased donor (DD) transplants increased by more than 33% from 1844 to 2465. Live donor transplant has decreased by 27% in recipients aged 18 to 34 years but increased by 21% in recipients older than 50 years.^1,2^ The reasons underlying this change are unclear.

There are potential explanations for this decrease in pediatric LD transplants. Deceased donor kidney allocation algorithms heavily prioritize pediatric transplant. At present, pediatric DD transplant rates are 40 per 100 patient wait-years compared with 15 per 100 patient waiting list–years in adults. Pediatric recipients also receive high-quality kidneys (75% receive a kidney with a kidney donor profile index of <20 and 95% with a kidney donor profile index of <35).^2^ Furthermore, some DD kidneys may be of higher quality than LD kidneys,^3^ and the ready availability of high-quality kidneys may be influencing the decision to opt for a DD kidney now to save an LD transplant for later.^4^ This strategy may have some benefit, given the longer waiting-list time and potential for lower-quality kidneys for those with a failed LD transplant who are wait-listed again in adulthood. In contrast, the potential LD allograft may no longer be available (medically unfit or the recipient sensitized), leading to a lost opportunity for transplant.

It is not clear whether the sequence of DD followed by LD (DD-LD) transplant is better than the sequence of LD followed by DD (LD-DD) transplant for long-term patient survival. However, both a lower availability of LD kidneys and deferred LD transplant rates may have considerable negative consequences for outcomes of pediatric patients with kidney disease. Therefore, the purpose of this medical decision analysis was to explore the conditions that would favor one sequence over the other.

## Methods

Research ethics approval and informed consent were waived by the Nova Scotia Health Authority Research Ethics Board for use of retrospective publicly available data. Reporting followed the Consolidated Health Economic Evaluation Reporting Standards (CHEERS) Checklist.^5^

### Model Structure

**Figure 1.**
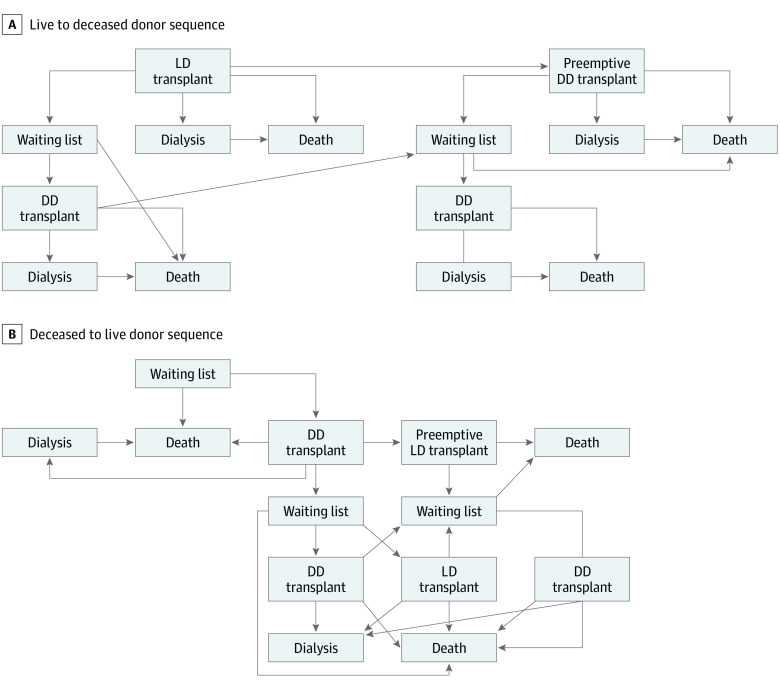
Pathways of the Live Donor (LD) to Deceased Donor (DD) and DD to LD Sequences

We used a decision analytic Markov model to compare 2 kidney transplant sequence options in recipients aged 3 to 25 years. The time horizon was until the recipient reached 90 years of age. In option 1 (LD-DD sequence), patients received an LD transplant without waiting. If this failed, they had the possibility of returning to the waiting list and obtaining a DD transplant (Figure 1A). In option 2 (DD-LD sequence), patients were initially on the waiting list, received a DD first, and if this transplant failed, received an LD transplant. For option 2, there was the risk that the LD would not be available. In this case, the patient would wait for a second DD transplant (Figure 1B). Both models included the possibility of a third DD transplant. For each option, we estimated total life-years and compared the difference in total life-years between options 1 and 2.

### Data Sources and Inputs

Annual mortality rates for waiting-listed patients undergoing dialysis, rates of death with a functioning transplant, and return to dialysis from a failed transplant were taken from unadjusted US Renal Data System Tables (2019 Report, eTable 1 in the Supplement).^6^ Annual rates of graft loss were calculated from 5-year probabilities of graft failure for LD and DD kidney transplants (eTable 2 in the Supplement). Baseline transplant rates for a DD kidney were 40 per 100 patient waiting list–years for pediatric recipients and 15 per 100 patient waiting list–years for adults (older than 18 years).^2^ Rates for a third transplant were 12 per 100 patient waiting list–years, and the transplant was assumed to be from a DD. The 2 sequence options were LD-DD and DD-LD; however, if the LD was no longer available, the sequence transformed to DD-DD. Rates were converted to transition probabilities.

### Model Assumptions

The model assumed a high probability (100%) of candidacy (medically eligible for another transplant) until 30 years of age. Thereafter, the probability of candidacy fell to 80% by 50 years of age, to 30% by 60 years of age, to 5% by 70 years of age, and to 0 (no option for a transplant) by 80 years of age (eTable 3 in the Supplement).^6,7,8,9^ Rates of preemptive transplant (another transplant before the transition to dialysis) after a failed first transplant were 20% for an LD and 15% for a DD transplant.^10,11^ At baseline, we assumed 10% of LD kidneys would not be available in the DD-LD sequence. We assumed the third transplant would not be preemptive. A detailed list of assumptions is outlined in eTable 4 in the Supplement.

### Sensitivity Analysis

In sensitivity analyses, we examined the following: (1) different probabilities regarding the availability of the LD transplant (DD-LD sequence), (2) different rates of DD transplant, (3) different probabilities of being a candidate for a second transplant, (4) different probabilities of receiving the second transplant preemptively, (5) lowering waiting-list mortality for patients aged 3 to 9 years (given that mortality rates among patients on the waiting list are very high for patients aged 3-9 years compared with patients aged 10-13 years^6^), and (6) having rates of LD graft loss 50% lower than the corresponding rates of DD graft loss for those aged 10 to 24 years (eTable 5 in the Supplement). We justified the latter factor because graft failure rates are highest from ages 14 to 25 years.^6^ Although rates of graft failure are lower for recipients of an LD compared with a DD kidney for all ages, the difference is not uniform. Graft loss rates are 40% to 60% lower for patients younger than 10 years and older than 25 years for LD compared with DD kidneys.^6^ However, rates of graft loss for LD transplants are only 20% to 40% lower compared with rates for DD graft loss in patients aged 10 to 24 years.

### Statistical Analysis

The Markov model was developed in TreeAge Pro Healthcare 2019, version R2.1 (TreeAge Software, LLC). The cycle length was yearly. Uncertainty in the net difference between options was examined by Monte Carlo microsimulation (1000 trials) to calculate 95% CIs.^12^ Log-normal distributions were applied to age-stratified patient mortality and graft failure transition probabilities.

## Results

**Figure 2.**
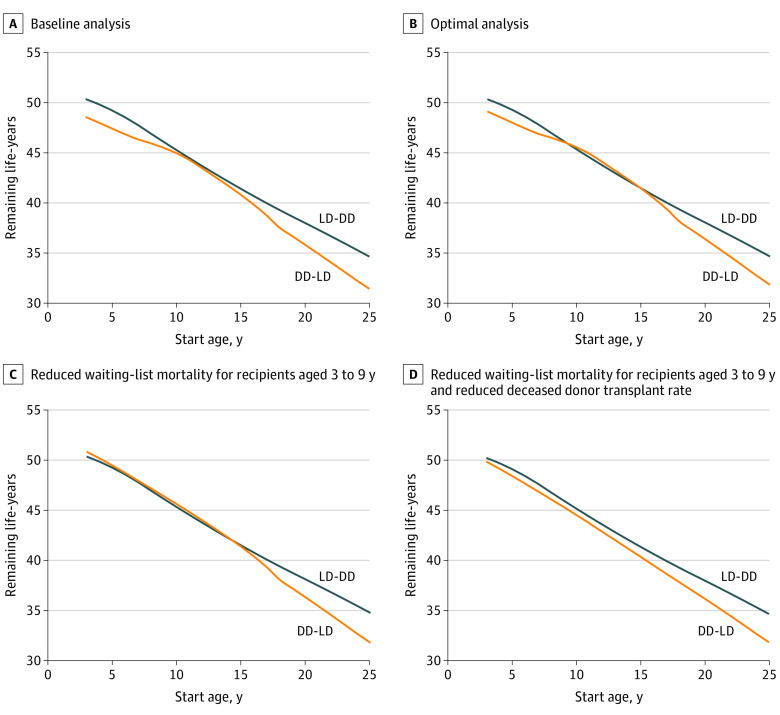
Remaining Life-Years by Age A, In the baseline analysis, the live donor to deceased donor (LD-DD) sequence provided more net life-years in patients 5 and 20 years of age compared with the DD-LD sequence. B, In the optimal analysis (LD is always available and preemptive), the DD-LD sequence improved relative to the LD-DD sequence, and the association between the 2 sequence outcomes was not consistent with starting age. C, In the optimal analysis and assuming reduced mortality for recipients aged 3 to 9 years, the DD-LD sequence was nearly equivalent to the LD-DD sequence for those aged 3 to 14 years. D, In the optimal assumptions, assuming reduced mortality for recipients aged 3 to 9 years and the DD transplant rate for recipients aged 3 to 20 years was set at the adult transplant rate, the differences between the 2 sequences were more uniform and in favor of the LD-DD sequence throughout.

The study population included US pediatric patients with kidney failure in the US Renal Data System 2019 Report^6^ who were waiting for a kidney transplant, received a transplant, or experienced graft failure. Data were analyzed from November 6, 2020, to June 1, 2021. Table 1 and Figure 2A show the outcomes between the 2 options across the range of ages. Under the baseline assumptions, the LD-DD sequence provided more net life-years in patients 5 years of age (1.82 [95% CI, 0.87-2.77]) and 20 years of age (2.23 [95% CI, 1.31-3.15]) compared with the DD-LD sequence. The net difference in life-years among those 10 years of age (0.36 [95% CI, −0.51 to 1.23]) and 15 years of age (0.64 [95% CI, −0.15 to 1.39]) was not significantly different. Table 1 and Figure 2B show how the DD-LD sequence improved relative to the LD-DD sequence if the LD kidney was always available and the transplant was preemptive. Under these conditions, the LD-DD sequence always remained superior for recipients younger than 10 years and older than 15 years, but at no time was the DD-LD sequence significantly better in those aged 10 to 14 years.

**Table 1.** Estimated Life-Years and Net Differences in Life-Years Depending on the Sequence of Kidney Transplants

Recipient age, y	Availability of LD in option 2	Option 1: LD-DD sequence		Option 2: DD-LD sequence		Net difference, life-years (95% CI)
		Life-years	No. of DD kidneys used	Life-years	No. of DD kidneys used	
3	Not available 10% of the time	50.32	0.74	48.53	1.27	1.80 (0.86 to 2.74)
	Always available			48.74	1.19	1.59 (0.73 to 2.55)
	Always available and preemptive recipient			49.06	1.22	1.26 (0.29 to 2.23)
5	Not available 10% of the time	49.22	0.73	47.40	1.26	1.82 (0.87 to 2.77)
	Always available			47.61	1.19	1.61 (0.71 to 2.51)
	Always available and preemptive recipient			47.91	1.21	1.31 (0.43 to 2.19)
10	Not available 10% of the time	45.3	0.7	44.94	1.26	0.36 (−0.51 to 1.23)
	Always available			45.16	1.19	0.14 (−0.72 to 1.00)
	Always available and preemptive recipient			45.47	1.21	−0.17 (−0.92 to 0.58)
15	Not available 10% of the time	41.45	0.61	40.81	1.16	0.64 (−0.15 to 1.39)
	Always available			41.01	1.11	0.44 (−0.26 to 1.14)
	Always available and preemptive recipient			41.37	1.13	0.08 (−0.57 to 0.73)
20	Not available 10% of the time	38.04	0.47	35.81	1.00	2.23 (1.31 to 3.15)
	Always available			35.96	0.96	2.09 (1.24 to 2.93)
	Always available and preemptive recipient			36.32	0.97	1.73 (0.83 to 2.63)
25	Not available 10% of the time	34.7	0.34	31.40	0.92	3.30 (2.40 to 4.22)
	Always available			31.51	0.89	3.19 (2.29 to 4.09)
	Always available and preemptive recipient			31.81	0.90	2.88 (1.98 to 3.78)

Abbreviations: DD, deceased donor; LD, live donor.

As shown in Figure 2A-B, the association between the 2 sequence outcomes was not consistent with starting age. Patients younger than 9 years always had better outcomes with the LD-DD sequence, even under optimal conditions for the DD-LD sequence (ie, the LD was always available and the transplant was preemptive). However, when waiting-list mortality rates for those aged 3 to 9 years were modeled to be the same as for those aged 10 to 13 years, the DD-LD option was nearly equivalent to the LD-DD sequence for those aged 3 to 14 years (Figure 2C).

Figure 2D shows how reducing the pediatric DD transplant rate to the adult rate under optimal conditions for the DD-LD sequence and lower waiting-list mortality for patients aged 3 to 9 years affected results. The differences between the 2 sequences were more uniform and in favor of the LD-DD sequence throughout.

**Figure 3.**
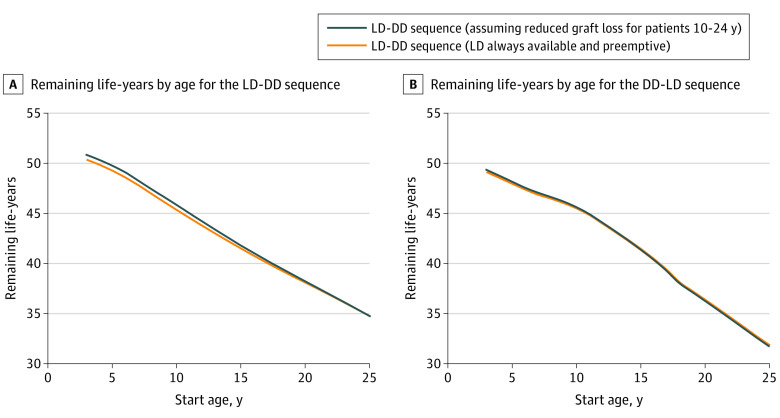
Remaining Life-Years by Age for Both Sequences A, The live donor–deceased donor (LD-DD) sequence with optimal assumptions (LD always available and preemptive) and under the assumption of reduced LD graft loss for patients aged 10 to 24 years. B, The DD-LD sequence with optimal assumptions (LD always available and preemptive) and under the assumption of reduced LD graft loss for patients aged 10 to 24 years.

Additional sensitivity analyses are shown in eTable 6 in the Supplement. For patients aged 3, 5, and 20 years, the LD-DD sequence was favored in all scenarios. For patients aged 10 years, favor for an LD-first sequence was statistically significant only if eligibility for a second transplant was low (2.09 [95% CI, 1.20-2.98] additional life-years). For patients aged 15 years, the LD-first sequence was favored if eligibility for a second transplant was low (1.84 [95% CI, 0.96-2.72] additional life-years), if the LD allograft was no longer available (2.49 [95% CI, 1.77-3.27] additional life-years), or if the pediatric DD transplant rate was low (1.07 [95% CI, 0.20-1.94] additional life-years). Reducing rates of LD graft loss by 50% relative to rates for DD graft loss from 10 to 24 years of age resulted in slightly better outcomes for the LD-DD sequence (Figure 3A) with little improvement in the DD-LD sequence (Figure 3B).

Table 2 explores the lost life-years if an LD was never available (DD-DD sequence) compared with the LD-DD sequence. For example, the model projects patients aged 10 years would lose 2.32 (95% CI, 1.52-3.12) life-years if there is no LD option. Table 2 also shows that reduced eligibility for a second graft accentuates the importance of an LD graft first. Although a third transplant option may be important for a given individual, eliminating the third transplant did not change the findings of the study.

**Table 2.** Estimated Life-Years and Net Differences in Life-Years Assuming No LD Is Available for a Second Transplant

Recipient age, y	Scenario	Option 1: LD-DD sequence		Option 2: DD-DD sequence		Net difference, life-years (95% CI)
		Life-years	No. of DD kidneys used	Life-years	No. of DD kidneys used	
3	1. Baseline assumptions	50.32	0.74	46.64	1.91	3.68 (2.78-4.58)
	2. 20% Lower candidacy after a failed transplant	47.85	0.55	43.35	1.66	4.11 (3.09-5.13)
	3. No third transplant option	49.26	0.48	45.40	1.61	3.86 (2.86-4.88)
5	1. Baseline assumptions	49.22	0.73	45.50	1.89	3.72 (2.71-4.74)
	2. 20% Lower candidacy after a failed transplant	46.77	0.55	42.65	1.66	4.12 (3.11-5.23)
	3. No third transplant option	48.19	0.48	44.32	1.60	3.87 (2.97-4.77)
10	1. Baseline assumptions	45.30	0.70	42.98	1.87	2.32 (1.52-3.12)
	2. 20% Lower candidacy after a failed transplant	42.93	0.53	40.27	1.66	2.66 (1.81-3.51)
	3. No third transplant option	44.34	0.47	41.95	1.61	2.38 (1.54-3.22)
15	1. Baseline assumptions	41.45	0.61	38.95	1.68	2.49 (1.71-3.27)
	2. 20% Lower candidacy after a failed transplant	39.38	0.46	36.77	1.51	2.61 (1.77-3.45)
	3. No third transplant option	40.70	0.43	38.27	1.51	2.43 (1.60-3.26)
20	1. Baseline assumptions	38.04	0.47	34.43	1.37	3.61 (2.70-4.52)
	2. 20% Lower candidacy after a failed transplant	36.48	0.36	33.07	1.26	3.41 (2.52-4.30)
	3. No third transplant option	37.60	0.35	34.13	1.28	3.47 (2.64-4.30)
25	1. Baseline assumptions	34.70	0.34	30.38	1.21	4.36 (3.53-5.19)
	2. 20% Lower candidacy after a failed transplant	33.61	0.26	29.44	1.13	4.17 (3.34-5.00)
	3. No third transplant option	34.46	0.27	30.23	1.15	4.23 (3.43-5.03)

Abbreviations: DD, deceased donor; LD, live donor.

Differences in the overall numbers of DD kidneys projected to be transplanted between the 2 sequences was also estimated. From 3 to 25 years of age, 0.45 to 0.55 more DD kidneys were used per recipient in the DD-LD group, assuming that an LD kidney was always available (Table 1). If the LD option was never used, the number of DD kidneys used was even higher for the DD-first sequence (Table 2).

## Discussion

Under most reasonable circumstances, the LD-first option maximizes recipient life-years. Nonetheless, using the LD-second option appears to be justified in recipients aged 10 to 14 years if an LD is always available and a transplant can always be performed preemptively or if recipients reside in a region with very low rates of adult transplant. Most importantly, this study found that a pediatric cohort with access to multiple DD kidneys would not, on average, outperform a cohort that also has access to LD kidneys.

This study also demonstrates the importance of the very high pediatric DD transplant rate. If pediatric rates of DD transplant were the same as in adults, then the benefit of a DD-LD sequence would always be inferior to the LD-DD scenario. Prioritization of DD kidney transplant for pediatric recipients has greatly improved patient survival but may have inadvertently reduced LD rates and remaining life-years.

This analysis also demonstrates the importance of waiting-list mortality. The benefit of a DD-first sequence is almost entirely realized by patients aged 10 to 14 years, because this is the group with the lowest waiting-list mortality. In contrast, the analysis shows that because of the relatively high waiting-list mortality of patients younger than 10 years, they are better served with an LD-first sequence. It is interesting to note that mortality rates for the general population and individuals with a functioning transplant tend to be lower from 5 to 9 years of age compared with individuals aged 10 to 13 years.^6,13^ The benefit of an LD-first sequence was negated if the mortality rates in the cohort of patients aged 3 to 9 years were reduced. The reasons for this finding are not clear, but this younger cohort may be more vulnerable to the rigors of dialysis; regardless, this finding requires further study. If this younger cohort is truly more vulnerable to dialysis, then even greater access to LD transplant through enhanced family support or even higher priority for DDs is needed. Some parents with very young children might have fewer supports to participate as LDs while being the primary caregiver for their child. The DD waiting list may be the only option. It may be that the overall trend for lower LD rates is a consequence of stricter eligibility criteria for LDs, particularly because donation is associated with an increased risk of kidney failure.^14^ Because parents may be having children at a later age, they may have more comorbidities that preclude donation. However, the registry data show that parent-to-child donation has fallen 46%, from 1823 transplants in 2001 through 2005 to 987 in 2015 through 2019, whereas parent-to–adult offspring donation has fallen only 30%, from 2377 to 1656 transplants.^1^

We examined the high rate of graft failure during adolescence to determine whether there were any associations between adolescence and the different sequences.^6,15,16^ In general, rates of graft failure are higher for DD compared with LD transplants for all patient ages; however, the rates are disproportionate at different ages.^6^ The rates of graft loss seem relatively high in LD compared with DD transplants during adolescence. High rates of graft loss could be associated with greater nonadherence to medication, poor transition from pediatric to adult care, physiologic stresses associated with development during adolescence, or recurrent disease.^15,16,17,18^ Smaller differences could be from the better-quality DD organs relative to LD kidneys in this age group, but that would not be consistent with the higher rates of graft loss and the disproportional differences seen in the younger pediatric cohorts. Interestingly, higher rates of graft loss during these years are not observed with liver transplants.^17^ Livers may tolerate lapses in immunosuppression due to nonadherence or may be better able to respond to the growing metabolic demands of adolescence than kidneys. Nonetheless, deferring the LD transplant to a time when outcomes are better is conceptually similar to deferring spending to a time of better purchasing power. Overall, the effect was smaller than we might have expected and benefits patients aged 3 to 13 years more than patients older than 14 years.

One of the more important unknowns is whether a pediatric patient will be eligible for a second transplant years later. The trend has been to refer, wait-list, and provide transplants to older recipients.^19^ However, information on the probability of a patient being medically eligible for retransplant is not well studied.^8,10,12,20,21,22^ Any reduction in later candidacy will affect the DD-LD sequence more than the LD-DD sequence.

Continuous and preemptive availability of an LD transplant is a worthy aspiration but is not always achievable, and this is an area for improvement for many transplant centers.^10,11^ Studies also show greater sensitization and lower rates of transplant in patients who undergo transplant with a DD kidney first compared with an LD kidney first, which further supports an LD-first sequence.^10,11^ Delays may be unavoidable (eg, precipitous graft loss, delay in LD workup or finding a new LD). For recipients who have developed HLA antibodies to their LD transplant, entering a paired exchange registry or desensitization are options. However, desensitization may be associated with inferior outcomes.^23^ On the other hand, an LD who was not available at the time of incident kidney failure may become available when the patient is older (eg, a family member, friend, or spouse). Having an LD allograft first also opens the possibility of an LD-LD sequence, which would provide even better outcomes (eTable 7 in the Supplement).

This is not the first study to examine whether the LD-DD or the DD-LD sequence is best. Van Arendonk et al^20^ concluded that an initial DD did not compromise the outcome of having an LD for the second transplant. However, some of these same authors reevaluated these options using a Markov model.^12^ Their model considered uncertainties of the LD being available and different transplant rates for a DD allograft. The second analysis found that under most circumstances, an LD-first sequence was best. However, they also found a DD-first sequence produced comparable patient survival if the recipients were highly sensitized. Although the model of Van Arendonk et al^12^ is similar to ours, our time horizon was extended to a lifetime, and the possibility of a third transplant was included. We also considered that not all patients with a failed transplant would be medically eligible for a second transplant and that the LD transplant might not be preemptive. Van Arendonk et al^12^ used percentage alive at year 20 as an outcome compared with our outcome of life-years. By our estimates for a transplant recipient aged 15 years, more than 85% of patients would still be alive 20 years later, 46% with an LD-first sequence would still have their first transplant, and only 37% with a DD-first sequence would have received an LD transplant.

### Limitations

There are limitations to our study. We assumed for the base case that 10% of the time an LD would not be available for the second transplant. Because the actual percentage is unknown and not easily predicted, we examined the entire range of possibilities. We assumed an LD would always be available sooner compared with a DD from the waiting list. This may not be true if the LD workup is delayed or prolonged. We used aggregate data by age groups (eg, 5 to 9 years); therefore, whether age 8 or 9 or 10 years is a better threshold for a change in option superiority is not certain. We also did not examine the effect of individual donor and recipient characteristics on outcomes.^12^ Individuals with reduced access to transplant, live donation, or preemptive listing will have worse outcomes in both sequences.^11^ We did not reduce transplant rates for a second transplant due to sensitization. Although sensitization will affect subsequent transplant rates, the current Kidney Allocation System has demonstrated an ability to transplant in those who are sensitized (≤98%) at a rate that is not very different from those less sensitized.^2^ We did not explore HLA mismatch effects. For example, recipients of HLA identical kidneys will likely have better survival and less subsequent sensitization if the graft fails regardless of when used.

## Conclusions

Understanding the relative benefits of one kidney transplant sequence compared with another is a complex combination of time-varying mortality for the different health states, rates of graft failure that differ by organ source, kidney transplant rates, candidacy for a second or third transplant, and probability that an LD available now will be suitable years later. Opting for an LD kidney first provides more net life-years under most scenarios; opting for a DD kidney first and delaying the LD to be a backup in a pediatric recipient is preferred only under a few narrow conditions. The novel aspect of this decision analytical model is that it allowed us to identify, isolate, and quantify the variables associated with this choice. Regardless, the reduction in LDs in this population during the last 20 years has and will have a significant negative effect on patient outcomes. Preemptive LD transplant should be vigorously pursued for all age groups.

## Notes

Supplement. **eTable 1.** Mortality Rates for the Health States per 1000 Patient-Years **eTable 2.** Converted Graft Failure Rates (Return to Dialysis or Retransplant) per 100 Patient-Years **eTable 3.** Retransplant Candidacy (Percentages Between Ages Interpolated) **eTable 4.** Model Description, Assumptions, Outcomes and Estimation of Uncertainty **eTable 5.** Reduced Live Donor Graft Loss Rate Ages 10 to 24 Years to 50% of Deceased Donor Rate Per 100 Patient-Years **eTable 6.** Additional Sensitivity Analyses **eTable 7.** Possibility of LD-LD
