# Supplementary material for: Optimal Sequencing of Deceased Donor and Live Donor Kidney Transplant Among Pediatric Patients With Kidney Failure
Source: JAMA Netw Open. 2022 Jan 6;5(1):e2142331. doi: 10.1001/jamanetworkopen.2021.42331 (PMC8739763; doi:10.1001/jamanetworkopen.2021.42331)
Supplement: Supplement. — eTable 1. Mortality Rates for the Health States per 1000 Patient-Years eTable 2. Converted Graft Failure Rates (Return to Dialysis or Retransplant) per 100 Patient-Years eTable 3. Retransplant Candidacy (Percentages Between Ages Interpolated) eTable 4. Model Description, Assumptions, Outcomes and Estimation of Uncertainty eTable 5. Reduced Live Donor Graft Loss Rate Ages 10 to 24 Years to 50% of Deceased Donor Rate Per 100 Patient-Years eTable 6. Additional Sensitivity Analyses eTable 7. Possibility of LD-LD [file jamanetwopen-e2142331-s001.pdf]

Supplementary Online Content

Kiberd BA, Vinson A, Acott PD, Tennankore KK. Optimal sequencing of deceased donor and live donor kidney transplant among pediatric patients with kidney failure. *JAMA Netw Open*. 2022;5(1):e2142331. doi:10.1001/jamanetworkopen.2021.42331

eTable 1. Mortality Rates for the Health States per 1000 Patient-Years

eTable 2. Converted Graft Failure Rates (Return to Dialysis or Retransplant) per 100 Patient-Years

eTable 3. Retransplant Candidacy (Percentages Between Ages Interpolated)

eTable 4. Model Description, Assumptions, Outcomes and Estimation of Uncertainty

eTable 5. Reduced Live Donor Graft Loss Rate Ages 10 to 24 Years to 50% of Deceased Donor Rate Per 100 Patient-Years

eTable 6. Additional Sensitivity Analyses

eTable 7. Possibility of LD-LD

This supplementary material has been provided by the authors to give readers additional information about their work.

© 2022 Kiberd BA et al. *JAMA Network Open*.

eTable 1. Mortality Rates for the Health States per 1000 Patient-Years[6]

Age	Wait List Rate (H.6)<br>Transition Probability<br>(2.5% to 97.5%)	Transplant (H.10)<br>Transition Probability<br>(2.5% to 97.5%)	Failed Transplant (H.7)<br>Transition Probability<br>(2.5% to 97.5%)
0-4	23.8<br>0.0235<br>(0.0165, 0.0352)	11.2<br>0.0111<br>(0.0164-0.0351)	No Data (input 22)<br>0.0218<br>(0.0152, 0.0326)
5-9	23.7<br>0.0234<br>(0.0164, 0.0351)	2.23<br>0.0022<br>(0.0016, 0.0033)	21.8<br>0.0216<br>(0.0151, 0.0323)
10-13	9.43<br>0.0094<br>(0.0066, 0.0141)	2.5<br>0.0025<br>(0.0017, 0.0037)	8.9<br>0.0089<br>(0.0062, 0.0133)
14-17	11.4<br>0.0113<br>(0.0079, 0.0170)	2.53<br>0.0025<br>(0.0018, 0.0038)	9.3<br>0.0093<br>(0.0065, 0.0139)
18-21	15.1<br>0.015<br>(0.0105, 0.0225)	4.27<br>0.0043<br>(0.003, 0.0064)	32.1<br>0.0316<br>(0.0221, 0.0474)
22-24	17.5<br>0.0173<br>(0.0121, 0.0260)	4.1<br>0.0041<br>(0.0029, 0.0061)	34.2<br>0.0336<br>(0.0235, 0.0504)
25-29	21.8<br>0.0216<br>(0.0151, 0.0323)	4.35<br>(0.0043<br>(0.003, 0.0065)	38.4<br>0.0377<br>(0.0264, 0.0565)
30-34	27.6<br>0.0272<br>(0.0191, 0.0408)	5.95<br>0.0059<br>(0.0042, 0.0089)	46.3<br>0.0452<br>(0.0317, 0.0679)
35-39	31.1<br>0.0306<br>(0.0214, 0.0459)	7.4<br>0.0074<br>(0.0052, 0.0111)	52.7<br>0.0513<br>(0.0359, 0.0770)
40-44	34.9<br>0.0343<br>(0.024, 0.0514)	9<br>0.009<br>(0.0052, 0.011)	63.9<br>0.0619<br>(0.0433, 0.0929)
45-49	43.0<br>0.0421<br>(0.0295, 0.0631)	13.1<br>0.013<br>(0.0091, 0.0195)	80.3<br>0.0772<br>(0.054, 0.1157)
50-54	49.1<br>0.479<br>(0.0335, 0.0719)	18<br>0.0178<br>(0.0125, 0.0268)	93.6<br>0.0894<br>(0.0625, 0.1340)
55-59	62.6<br>0.0607<br>(0.0425, 0.091)	26.1<br>0.0258<br>(0.018, 0.0386)	129<br>0.121<br>(0.0847, 0.1815)
60-64	71.5<br>0.069<br>(0.0483, 0.1035)	36.1<br>0.0355<br>(0.0248, 0.0532)	159<br>0.147<br>(0.1029, 0.2205)
65-69	88.8	51.9	201

© 2022 Kiberd BA et al. *JAMA Network Open*.

	0.085<br>(0.0595, 0.1275)	0.0506<br>(0.0354, 0.0759)	0.182<br>(0.1275, 0.2731)
70-74	102<br>0.097<br>(0.0679, 0.1455)	72.8<br>0.0702<br>(0.0491, 0.1053)	249<br>0.22<br>(0.1543, 0.3306)
75-79	108.6<br>0.1029<br>(0.072, 0.1544)	103<br>0.0979<br>(0.0685, 0.1468)	307<br>0.2643<br>(0.185, 0.395)
80-84	136.8<br>0.1279<br>(0.0895, 0.1918)	142<br>0.134<br>(0.0927, 0.1986)	351<br>0.296<br>(0.207, 0.444)
85+	198.5<br>0.18<br>(0.126, 0.2701)	171<br>0.157<br>(0.126, 0.27)	311<br>0.2673<br>(0.187, 0.401)

Rates were the average of 2014 to 2017 inclusive (see corresponding USRDS unadjusted tables H.6, H.10, H.7). Rates were converted to annual mortality probabilities for the Markov transitions. The dispersions (probability densities) were lognormal with the approximate 2.5% to 97.5% depicted for each grouping [TreeAge interpolates rates at each year and each run will have a slightly different dispersion].

From a previous analysis, the hazard ratio for mortality in a wait listed patient age 0 to 11 (reference age 50 to 59) is 0.45 (95% CI; 0.34, 0.60). The 2.5% and 97.5% values for our lognormal distribution and would translate to hazard ratio of 0.45 would be 0.30 to 0.67. (Massie AB, Boyarsky BJ, Werbel WA, et al. Identifying scenarios of benefit or harm from kidney transplantation during the COVID-19 pandemic: A stochastic simulation and machine learning study. Am J Transplant. 2020 Nov;20(11):2997-3007).

© 2022 Kiberd BA et al. *JAMA Network Open*.

eTable 2. Converted Graft Failure Rates (Return to Dialysis or Retransplant) per 100 Patient-Years[6]

Age	Live Donor (F.23)<br>5 Year Probability of Return to Dialysis or Retransplant	Live Donor<br>Converted Rate of Graft Loss per 100 patient years<br>Transition Prob<br>(2.5% to 97.5%)	Deceased Donor (F.17)<br>5 Year Probability of Return to Dialysis or Retransplant	Deceased Donor<br>Converted Rate of Graft Loss per 100 patient years<br>Transition Prob<br>(2.5% to 97.5%)
0-4	6.70	1.39<br>0.0139<br>(0.0097, 0.0174)	11.70	2.49<br>0.02487<br>(0.0174, 0.0373)
5-9	4.42	0.91<br>0.0090<br>(0.0063, 0.01359)	12.10	2.57<br>0.0253<br>(0.0177, 0.0381)
10-13	10.80	2.29<br>0.0226<br>(0.0158, 0.0339)	13.40	2.88<br>0.0283<br>(0.0199, 0.0425
14-17	18.90	4.19<br>0.0410<br>(0.0287, 0.0615)	25.80	5.97<br>0.0579<br>(0.0405, 0.0965)
18-21	20.40	4.56<br>0.0446<br>(0.0312, 0.0668)	31.40	7.54<br>0.0726<br>(0.0508, 0.109)
22-24	18.00	3.97<br>0.0389<br>(0.0273, 0.0450)	28.30	6.65<br>0.0643<br>(0.0453, 0.0965)
25-29	15.40	3.34<br>0.0328<br>(0.0272, 0.0583)	23.30	5.31<br>0.0517<br>(0.0362, 0.0775)
30-34	11.00	2.34<br>0.0231<br>(0.0162, 0.0347)	18.30	4.04<br>0.0396<br>(0.0277, 0.0594)
35-39	10.50	2.34<br>0.0231<br>(0.0162, 0.0347)	17.70	3.90<br>0.0382<br>(0.0268, 0.0573)
40-44	8.50	1.77<br>0.0175<br>(0.01228, 0.0263)	15.40	3.34<br>0.0309<br>(0.0216, 0.0463)
45-49	7.52	1.56<br>0.0155<br>(0.0108, 0.0232)	14.50	3.14<br>0.0309<br>(0.02163, 0.0463)
50-54	6.80	1.41<br>0.014<br>(0.0098, 0.021)	12.70	2.72<br>(0.0268<br>(0.01878, 0.0403)
55-59	6.78	1.40<br>0.0139<br>(0.0097, 0.02085)	13.3	2.86<br>0.0282<br>(0.01974, 0.04229)
60-64	5.88	1.21<br>0.012<br>(0.0084, 0.01814)	12.5	2.66<br>0.0263<br>(0.0184, 0.0394)
65-69	5.53	1.14	13.0	2.79

© 2022 Kiberd BA et al. *JAMA Network Open*.

		0.0113<br>(0.0079, 0.017)		0.0275<br>(0.0193, 0.0412)
70-74	6.38	1.32<br>0.0131<br>(0.00918, 0.01967)	13.4	2.87<br>0.0283<br>(0.0198, 0.03995)
75-79	5.63	1.12<br>0.0111<br>(0.007796, 0.0167)	12.6	2.7<br>0.0266<br>(0.01864, 0.03995)

Graft failure rates were averaged over the most recent 4 years (2009 to 2012) from the 5-year unadjusted Tables (F.17 and F.23). Rates were converted to annual mortality probabilities for the Markov transitions. The dispersions (probability densities) were lognormal with the approximate 2.5% to 97.5% depicted for each grouping [TreeAge interpolates rates at each year and each run will have a slightly different dispersion].

Graft failure (LD and DD) was lognormal with an override mean of 1 and sigma of 0.2. The 2.5% and 97.5% values for the distribution were approximately 0.7 and 1.5 and mapped to a Kidney Donor Profile Index (https://optn.transplant.hrsa.gov/media/1512/guide_to_calculating_interpreting_kdpi.pdf accessed May 30, 2021) of approximately 5% and 80% respectively. This also corresponded to a Living Kidney Donor score of -20 to 60 respectively.[3]

Uncertainty in transplant rates were examined by a gamma distribution (2.5% to 97.5%). For pediatric DD transplant rates 40 per 100-patient-wait list years (26.8, 57.6) and for adult DD transplant rates 15 per 100-patient-wait list years (13.4, 28.8). Given even larger differences between Donor Service Areas even larger ranges were explored.[2] The 5-year DD cumulative transplant incidence varies from 15.5% to 67.8%. These would correlate approximately to rates of 3.3 to 25 DD transplant per 100 patient wait years by the end of 5 years. Therefore, in the sensitivity analysis we examined adult DD rates as low as 6 and pediatric rates as high as 70 per 100-patient-wait list years. 5-year cumulative incidence rates for pediatric recipients by area is not available.

© 2022 Kiberd BA et al. *JAMA Network Open*.

eTable 3. Retransplant Candidacy (Percentages Between Ages Interpolated)

Age	Baseline	20% reduced	50% Reduced
To 30	100%	80%	50%
50	80%	64%	40%
60	30%	24%	15%
70	5%	4%	2.5%
80	0%	0%	0%

There was no distribution sampling for this input only a one-way sensitivity analysis for probabilities above.

© 2022 Kiberd BA et al. *JAMA Network Open*.

eTable 4. Model Description, Assumptions, Outcomes and Estimation of Uncertainty

**Factor**	**Description**	**Location**
**Model Description**	Pediatric recipients with an available and eligible live donor could chose between 2 options. Option 1 would be a live donor (LD) first followed by a deceased donor (DD). Option 2 would be a DD first followed by a LD.	N/A
**Assumptions**	*Option 1(LD-DD) assumptions.*<br>1. The patient would receive a LD (first transplant) immediately. Over time, this LD could fail or the patient could die with a functioning LD transplant.<br>2. If the LD transplant failed, the patient could either A. receive a pre-emptive DD transplant, B. transition to dialysis and be placed on the wait list for a DD organ, or C. if the patient was no longer eligible for a transplant (medically unfit) remain on permanent dialysis until death.<br>3. If the patient received a DD (second transplant), this transplant could fail or they could die with a functioning transplant.<br>4. If the DD transplant failed, the patient could be placed on the wait list for another DD transplant (if eligible), or if not eligible, remain on permanent dialysis until death.<br>5. If they received a DD (third transplant), they could eventually die with a functioning transplant or return to permanent dialysis until death.<br>6. The analysis was truncated at age 90. The cycles were yearly.<br><br>*Option 2 (DD-LD) assumptions.*<br>1. The patient would be placed the pediatric wait list. If they survived the wait list, they would receive a DD (first transplant). Over time, this DD could fail or the patient could die with a functioning DD transplant.<br>2. If the DD transplant failed, the patient could receive a pre-emptive LD (second transplant) if the patient still had a LD available or receive a LD after transitioning to dialysis and while being on the wait list. If a LD was not available the patient could receive a pre-emptive DD transplant, could transition to dialysis and be placed on the wait list for a DD (second	N/A

© 2022 Kiberd BA et al. *JAMA Network Open*.

	transplant), or if the patient was no longer eligible for a transplant remain on dialysis until death.<br>3. If the patient received either an LD (second transplant) or a DD (second transplant), this transplant could fail or they could die with a functioning transplant.<br>4. If the second transplant failed, they could transition to dialysis and be placed on the wait list for another DD (third transplant) if eligible, or go on permanent dialysis until death.<br>5. If they received a DD (third transplant), they could eventually die with a functioning transplant or return to permanent dialysis until death.<br>6. The analysis was truncated at age 90. The cycles were yearly.<br><br>*Other assumptions for both options.*<br>1. Eligibility for a repeat transplant was age dependent and was reduced as the patient became older, and was no longer an option above age 80.<br>2. Transplantation rates from the wait list were dependent on the patient age (higher for pediatric than for adults).<br>3. Patients with a LD had better graft survival than those with a DD and graft survival was age dependent.<br>4. Patient survival was age dependent and mortality increased with age.<br>5. Patient who failed a transplant had higher rates of death than those who were eligible for a repeat transplant (wait list).	
**Inputs**	1. Mortality Rates<br>2. Graft Failure Rates for live and deceased donors<br>3. Candidacy Rates (eligibility for a repeat transplant. Since no data is available on candidacy rates, a range was explored	eTable 2<br>eTable 3<br>eTable 4
**Outcomes**	1. Remaining life years for each option.<br>2. The difference in remaining life years between the options.	N/A
**Estimating Uncertainty**	1. Transition probabilities: Rates were converted to probabilities<br>2. Distribution/Dispersion: Probabilities were multiplied by a lognormal	eTable 3/4

© 2022 Kiberd BA et al. *JAMA Network Open*.

	distribution (overriding mean 1 and sigma 0.2) to correspond to observed relative risks in patient mortality and LD and DD graft failure. A gamma distribution was used for annual DD transplantation transition probabilities. Since there is a large variation in transplant rates between Donor Service Areas a wider range was explored.<br>3. Confidence intervals for the net difference between options: A microsimulation of 1000 counterfactual trials were run to calculate 95% confidence intervals (mean±1.96*SE) as used in a similar Markov pediatric transplant model (12). Higher trial simulations to 10,000 would reduce the confidence interval by approximately 30%.	

© 2022 Kiberd BA et al. *JAMA Network Open*.

eTable 5. Reduced Live Donor Graft Loss Rate Ages 10 to 24 Years to 50% of Deceased Donor Rate Per 100 Patient-Years

Age	Deceased Donor Transplant Rate	Original Live Donor Graft Loss Rate	Reduced Live Donor Transplant Rate (50% of Deceased Donor Rate)
10-13	2.88	2.29	1.44
14-17	5.97	4.19	2.98
18-21	7.54	4.56	3.77
22-24	6.65	3.97	3.33

© 2022 Kiberd BA et al. *JAMA Network Open*.

eTable 6. Additional Sensitivity Analyses

Recipient Age	Scenario	Option 1: Live donor followed by deceased donor<br><br>Life years	Option 2: Deceased donor followed by live donor<br><br>Life years	Difference Option 1-Option 2<br><br>Life years (95% CI)
3 years	Baseline (B)	50.32	48.53	1.80 (0.86, 2.74)
	B+ 50% (vs. 0%) Reduced Regraft Candidacy	46.72	43.84	3.59 (2.59, 4.59)
	B+ 20% (vs. 10%) LD not Available (DD-LD)*	50.32	48.32	2.00 (1.09, 2.91)
	B+ 60 (vs. 40) per 100 Ped DD Transplant Rate	50.35	49.05	1.30 (0.37, 2.27)
	B+ 25 (vs. 40) per 100 Ped DD Transplant Rate	50.28	47.73	2.56 (1.53, 3.59)
	B+ 6 (vs. 15) per 100 Adult DD Transplant Rate	49.72	48.44	1.28 (0.22, 2.34)
	B+ 25 (vs. 15) per 100 Adult DD Transplant Rate	50.55	48.56	1.99 (1.06, 2.92)
	B+ No (vs. 0.15) pre-emptive DD (LD-DD)	50.19	48.53	1.66 (0.61, 2.71)
	B+ 50 (vs. 70) per 100 LD rate (DD-LD)	50.32	48.46	1.87 (0.90, 2.77)
	B+ 25% higher graft loss with repeat transplant	49.92	47.99	1.93 (0.95, 2.91)
5 years	Baseline (B)	49.22	47.40	1.82 (0.87, 2.77)
	B+ 50% Reduced Regraft Candidacy	45.64	42.76	3.59 (2.57, 4.61)
	B+ 20% LD not Available	49.22	47.19	2.03 (1.09, 2.97)
	B+ 60 per 100 Ped DD Transplant Rate	49.25	47.86	1.39 (0.49, 2.29)
	B+ 25 per 100 Ped DD Transplant Rate	49.19	46.73	2.47 (1.42, 3.52)
	B+ 6 per 100 Adult DD Transplant Rate	48.60	47.31	1.29 (0.35, 2.23)
	B+ 25 per 100 Adult DD Transplant Rate	49.46	47.44	2.03 (0.98, 3.08)
	B+ No pre-emptive DD (LD-DD)	49.09	47.40	1.69 (0.67, 2.71)
	B+ 50 (vs 70) per 100 LD rate (DD-LD)	49.22	47.33	1.89 (0.95, 2.83)
	B+ 25% higher graft loss with repeat transplant	48.82	46.88	1.95 (1.01, 2.89)
10 years	Baseline (B)	45.30	44.94	0.36 (-0.51, 1.23)
	B+ 50% Reduced Regraft Candidacy	41.84	40.45	2.09 (1.20, 2.98)
	B+ 20% LD not Available	45.30	44.72	0.58 (-0.22, 1.38)
	B+ 60 per 100 Ped DD Transplant Rate	45.32	45.16	0.16 (-0.61, 0.93)

© 2022 Kiberd BA et al. *JAMA Network Open*.

	B+ 25 per 100 Ped DD Transplant Rate	45.28	44.53	0.75 (-0.08, 1.58)
	B+ 6 per 100 Adult DD Transplant Rate	44.79	44.63	0.16 (-0.69, 1.01)
	B+ 25 per 100 Adult DD Transplant Rate	45.56	45.50	0.56 (-0.25, 1.37)
	B+ No pre-emptive DD (LD-DD)	45.16	44.94	0.22 (-0.59, 1.03)
	B+ 50 (vs 70) per 100 LD rate (DD-LD)	45.30	44.86	0.44 (-0.37, 1.25)
	B+ 25% higher graft loss with repeat transplant	44.92	44.46	0.46 (-0.32, 1.24)
15 years	Baseline (B)	41.45	40.81	0.64 (-0.15, 1.39)
	B+ 50% Reduced Regraft Candidacy	38.41	37.07	1.84 (0.96, 2.72)
	B+ 20% LD not Available	41.45	40.60	0.85 (-0.01, 1.71)
	B+ 60 per 100 Ped DD Transplant Rate	41.45	41.15	0.30 (-0.52, 1.22)
	B+ 25 per 100 Ped DD Transplant Rate	41.44	40.37	1.07 (0.20, 1.94)
	B+ 6 per 100 Adult DD Transplant Rate	40.69	40.30	0.38 (-0.58, 1.34)
	B+ 25 per 100 Adult DD Transplant Rate	41.74	41.00	0.74 (-0.06, 1.54)
	B+ No pre-emptive DD (LD-DD)	41.29	40.81	0.49 (-0.39, 1.29)
	B+ 50 (vs 70) per 100 LD rate (DD-LD)	41.45	40.72	0.73 (-0.15, 1.61)
	B+ 25% higher graft loss with repeat transplant	41.13	40.44	0.69 (-0.11, 1.59)
20 years	Baseline (B)	38.04	35.81	2.23 (1.31, 3.15)
	B+ 50% Reduced Regraft Candidacy	35.75	33.40	2.47 (1.53, 3.41)
	B+ 20% LD not Available	38.04	35.65	2.39 (1.44, 3.34)
	B+ 60 per 100 Ped DD Transplant Rate	38.04	35.81	Baseline
	B+ 25 per 100 Ped DD Transplant Rate	38.04	35.81	Baseline
	B+ 6 per 100 Adult DD Transplant Rate	37.28	33.76	3.52 (2.42, 4.58)
	B+ 25 per 100 Adult DD Transplant Rate	38.35	36.56	1.75 (0.89, 2.61)
	B+ No pre-emptive DD (LD-DD)	37.88	35.81	2.07 (1.23, 2.91)
	B+ 50 (vs 70) per 100 LD rate (DD-LD)	38.04	35.73	2.32 (1.42, 3.22)
	B+ 25% higher graft loss with repeat transplant	37.81	35.60	2.21 (1.26, 3.16)

*LD; Live Donor, DD; Deceased Donor, LD-DD; Live Donor for first transplant followed by deceased donor, DD-LD; Deceased donor for first transplant followed by live donor

© 2022 Kiberd BA et al. *JAMA Network Open*.

eTable 7. Possibility of LD-LD*

Age	LD-LD years	DD-LD years	Difference (LD-LD)-(DD-LD) years
3	51.97	48.53	3.44 (2.58, 4.30)
5	50.87	47.40	3.47 (2.55, 4.39)
10	46.94	44.94	2.00 (1.09, 2.91)
15	43.03	40.81	2.22 (1.32, 3.12)
20	39.42	35.81	3.61 (2.75, 4.47)

*Assumptions: 1. second LD is 20% pre-emptive. 2. 10% sensitized and only eligible for first DD (if still a candidate). 3. Also includes possibility of a third deceased donor transplant. Abbreviations: LD; Live Donor, DD; Deceased Donor

© 2022 Kiberd BA et al. *JAMA Network Open*.
